# Supplementary material for: Thermodynamics of deposition flux-dependent intrinsic film stress
Source: Nat Commun. 2016 Feb 18;7:10733. doi: 10.1038/ncomms10733 (PMC4759625; doi:10.1038/ncomms10733)
Supplement: Supplementary Information — Supplementary Figures 1-5, Supplementary Notes 1-3 and Supplementary References. [file ncomms10733-s1.pdf]

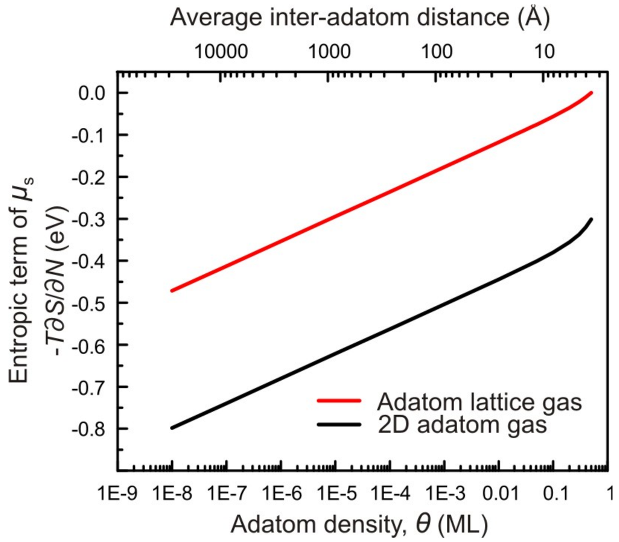

Supplementary Figure 1 | Entropic term of the surface chemical potential. The values are calculated for a Cu adatom gas on a Cu(111) surface using Adatom Lattice Gas model and the 2D Adatom Gas model. Please note that for all coverages below 0.1 ML, the slopes of the two different models are practically the same. This also means that both models deliver the same result as long as only variations in the chemical potential $\Delta\mu_s$ with respect to the adatom density are of importance.

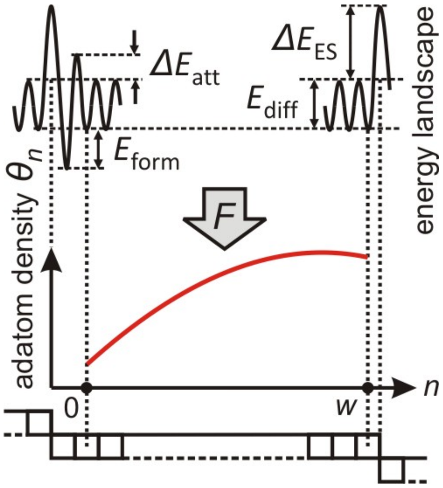

Supplementary Figure 2 | Calculated adatom density and energy landscape of a terrace. θ_n corresponds to adatom density at the $(n+1)^{\text{th}}$ site away from the ascending step on a terrace with width *w* taking into account realistic formation and diffusion barriers as indicated by the energy landscape at the top.

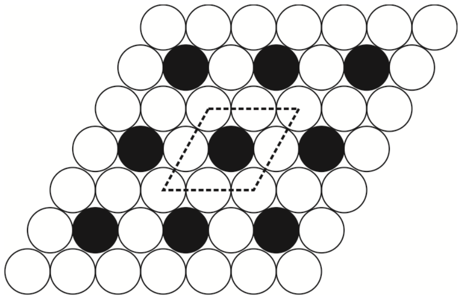

Supplementary Figure 3 | Ball model of a closed packed surface showing the maximum possible adatom density ($\theta_n = 0.25$). White circles correspond to the available surface sites and black circles represent adatoms. If one would place one additional adatom on this surface, an adatom island is formed. In this sense the maximum meaningful density for individual, single adatoms on a close packed surface is given by $\theta_n = 0.25$.

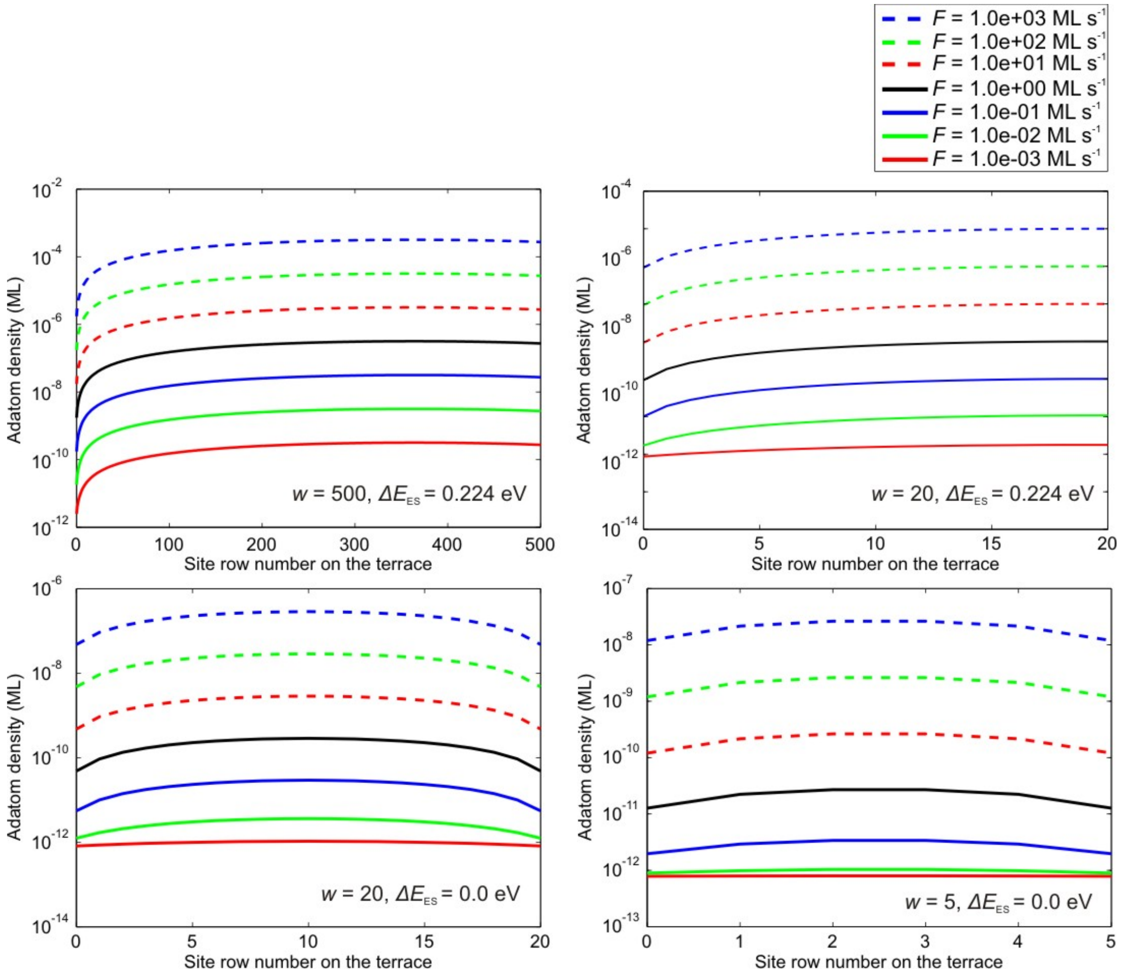

Supplementary Figure 4 | Adatom density profiles for terraces with different widths. The curve are calculated for Cu(111) terraces with different width (w) as well as the presence and absence of an Ehrlich-Schwoebel barrier (ΔE_{ES}). The lower two panels describe the funneling situation in the vicinity of the GB's.

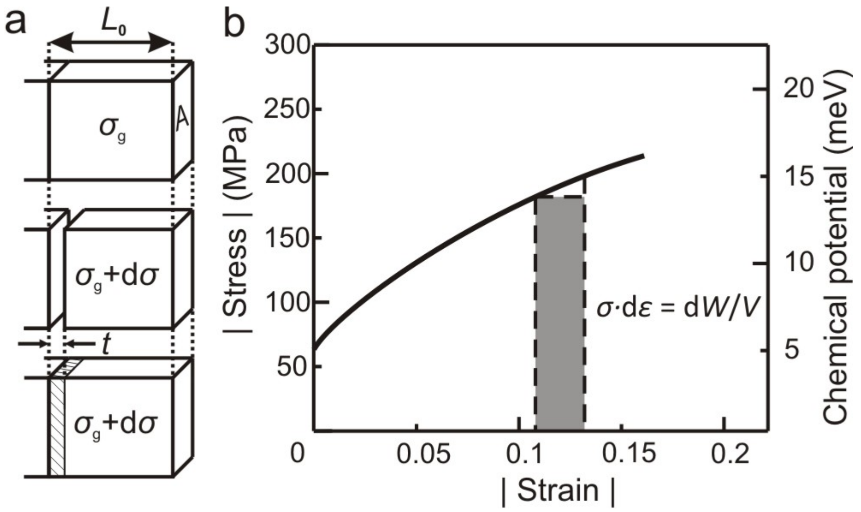

Supplementary Figure 5 | Relation between the chemical potential and stress within a grain. a, adding a thin layer to a pre-stressed grain. **b,** The compressive strain-stress curve for copper, reproduced from Ref. [1]. For small strain values the deformation work can be approximated by the shaded area. Here, the width of the shaded area is exaggerated for clarity. The right vertical axis shows the chemical potential values corresponding to the stress values on the left. The red circles correspond to the reported reversible stress values in the literature. Refer to the caption of supplementary figure 3 in the main manuscript for the references.

Supplementary Note 1 - Chemical potential of surface adatom gas

Adatom Lattice Gas (ALG) model

In this model the adatoms are assumed to reside in discrete lattice sites on the surface [2]. Imagine a terrace that has N_{site} possible number of sites, where adatoms want to rest. If one has to put N_{ad} adatoms on this surface, the number of different configurations can be calculated as:

$$\Omega = \frac{N_{\text{site}}!}{N_{\text{ad}}!\left(N_{\text{site}} - N_{\text{ad}}\right)!} \qquad (1)$$

From this we can derive the entropy of this particular adatom gas on this particular surface:

$$S = k_{\text{B}} \ln \frac{N_{\text{site}}!}{N_{\text{ad}}!\left(N_{\text{site}} - N_{\text{ad}}\right)!}$$

, and with the Stirling's approximation we derive:

$$S = k_{\text{B}} \left[\ln\left(N_{\text{site}}!\right) - \left(N_{\text{ad}} \ln N_{\text{ad}} - N_{\text{ad}}\right) - \left(N_{\text{site}} - N_{\text{ad}}\right) \ln\left(N_{\text{site}} - N_{\text{ad}}\right) + \left(N_{\text{site}} - N_{\text{ad}}\right) \right]$$

Therefore we can write the entropic term of the chemical potential of the adatom gas as:

$$-T \frac{\partial S}{\partial N_{\text{ad}}} = -k_{\text{B}} T \ln\left(\frac{N_{\text{site}} - N_{\text{ad}}}{N_{\text{ad}}}\right) \qquad (2)$$

, and by defining $\theta = N_{\text{ad}} / N_{\text{site}}$ we end at:

$$\boxed{\left(-T \frac{\partial S}{\partial N_{\text{ad}}}\right)_{\text{ALG}} = k_{\text{B}} T \ln\left(\frac{\theta}{1-\theta}\right)} \qquad (3)$$

Two Dimensional Adatom Gas (2DAG) model

In this model the adatoms are assumed to be completely delocalized and to behave as "*true*" particles of a 2D gas on the surface [2]. The partition function of an adatom gas containing N_{ad} adatoms is the multiple of the partition functions of each adatom divided by $N_{\text{ad}}!$. The division by $N_{\text{ad}}!$ removes the double counting of the states:

$$Z_{\text{2DAG}} = \frac{Z_{\text{ad}}^{\;N_{\text{ad}}}}{N_{\text{ad}}!} \qquad (4)$$

The partition functions of a single adatom on a 2D surface with only in-plane (X and Y) degrees of freedom can be written as:

$$Z_{\mathrm{ad}} = \sum_{k_x}\sum_{k_y} \exp\left(-\frac{E}{k_B T}\right) = \sum_{k_x}\sum_{k_y} \exp\left(-\frac{\frac{\hbar^2}{2m}\left(k_x{}^2 + k_y{}^2\right)}{kT}\right) = \sum_{k_x}\sum_{k_y} \exp\left(-\frac{\hbar^2}{2mk_B T}\left(\left(\frac{2\pi n_x}{L_x}\right)^2 + \left(\frac{2\pi n_y}{L_y}\right)^2\right)\right)$$

, in which we used the quantum mechanical description of particle in a box in a 2 dimensional space. By assuming this space to be macroscopic in size and with $L_x = L_y = L$, this results in:

$$Z_{\mathrm{ad}} = \sum_{k_x=-\infty}^{+\infty}\sum_{k_y=-\infty}^{+\infty} \exp\left(-\frac{h^2\left(n_x{}^2 + n_y{}^2\right)}{2mk_B TL^2}\right) = \int_{x=-\infty}^{+\infty}\int_{y=-\infty}^{+\infty} \exp\left(-\frac{h^2\left(x^2 + y^2\right)}{2mk_B TL^2}\right) =$$

$$= \int_{x=-\infty}^{+\infty} \exp\left(-\frac{h^2 x^2}{2mk_B TL^2}\right)\int_{y=-\infty}^{+\infty} \exp\left(-\frac{h^2 y^2}{2mk_B TL^2}\right) = \left(\sqrt{\pi}\,\frac{\sqrt{2mk_B T}L}{h}\right)^2 = \frac{L^2 2\pi mk_B T}{h^2}$$

This can be written as:

$$Z_{\mathrm{ad}} = \frac{A_{\mathrm{eff}}}{\Lambda^2} \qquad (5)$$

, in which $\Lambda = \frac{h}{\sqrt{2\pi mk_B T}}$ is the thermal de Broglie wavelength, and A_{eff} is the effective available surface area that obviously decreases the more adatoms are present on the surface. For a single adatom A_{eff} is evidently equal to the total surface area $A_{\mathrm{eff}} = A_{\mathrm{surf}} = L^2$. By combining Eqs. 4 and 5 the total partition function of a 2DAG becomes:

$$Z_{\mathrm{2DAG}} = \frac{\left(A_{\mathrm{eff}} / \Lambda^2\right)^{N_{\mathrm{ad}}}}{N_{\mathrm{ad}}!} \qquad (6)$$

The entropy of this 2DAG can be derived as:

$$S = k_B \ln Z_{\mathrm{2DAG}} = k_B\left[N_{\mathrm{ad}} \ln\left(A_{\mathrm{eff}} / \Lambda^2\right) - N_{\mathrm{ad}} \ln N_{\mathrm{ad}} + N_{\mathrm{ad}}\right] = k_B\left[N_{\mathrm{ad}} \ln\left(\frac{A_{\mathrm{surf}} - N_{\mathrm{ad}}A_{\mathrm{ad}}}{\Lambda^2 N_{\mathrm{ad}}}\right) + N_{\mathrm{ad}}\right]$$

In the last step we used $A_{\mathrm{eff}} = A_{\mathrm{sur}} - N_{\mathrm{ad}}A_{\mathrm{ad}}$.

The entropic component of the chemical potential of 2DAG is therefore given by:

$$-T\frac{\partial S}{\partial N_{\mathrm{ad}}} = -k_B T\left[\ln\left(\frac{A_{\mathrm{sur}} - N_{\mathrm{ad}}A_{\mathrm{ad}}}{\Lambda^2 N_{\mathrm{ad}}}\right) - \frac{N_{\mathrm{ad}}A_{\mathrm{ad}}}{A_{\mathrm{sur}} - N_{\mathrm{ad}}A_{\mathrm{ad}}}\right] \qquad (7)$$

By defining $\theta = N_{\mathrm{ad}}A_{\mathrm{ad}} / A_{\mathrm{sur}}$ i.e. replacing $N_{\mathrm{ad}} = \theta A_{\mathrm{sur}} / A_{\mathrm{ad}}$ and further simplifying one gets:

$$\left(-T\frac{\partial S}{\partial N_{\mathrm{ad}}}\right)_{\mathrm{2DAG}} = k_{\mathrm{B}}T\left[\ln\left(\frac{\Lambda^2}{A_{\mathrm{ad}}}\frac{\theta}{1-\theta}\right)+\frac{\theta}{1-\theta}\right] \tag{8}$$

Entropic component of the chemical potential: ALG vs 2DAG

The thermodynamical derivation in the paper depends crucially on the entropic term of the chemical potential of the surface. Therefore, we plot in supplementary figure 1 $-T(\partial S / \partial N_{\mathrm{ad}})$ for both the ALG as well as the 2DAG taking into account the particular values for Cu adatoms on a Cu(111) surface.

Supplementary Note 2 - Adatom density at specific sites on a terrace

Supplementary figure 2 shows a simplified cross sectional model of the film surface, in which we define the position of the first lattice row next to the ascending step edge as the origin of a terrace with width w. The adatom density at the $(n+1)^{\mathrm{th}}$ site away from ascending step edge (θ_n) can be derived via the general differential equation for mass conservation on the terrace:

$$\frac{\partial \theta_n}{\partial t} = \nu_{\mathrm{d}} \frac{\partial^2 \theta_n}{\partial n^2} + V_{\mathrm{s}} \frac{\partial \theta_n}{\partial n} - \nu_{\mathrm{e}} \theta_n + F \qquad (9)$$

, in which ν_{d} is the diffusion rate of adatoms, V_{s} the step growth speed, ν_{e} the evaporation rate of adatoms from the terrace into the vacuum, and F the deposition rate.

Here we have used a dimensionless definition of the adatom density, i.e. θ_n is the adatom coverage in monolayers [ML] and F has the unit ML s^{-1}. This implies that $\theta_n = 0$ corresponds to a surface with zero adatoms and $\theta_n = 1$ to a fully covered surface with a complete monolayer of adatoms. Please note that for the case of homoepitaxy (deposited material is the same as the substrate material), the states with $\theta_n = 0$ and $\theta_n = 1$ describe exactly the same surface state. If one considers only individual adatoms (and no formation of adatom islands), the maximum meaningful adatom density is given by $\theta_n \le 0.25$ for a close packed surface, as is illustrated in supplementary figure 3.

At a certain temperature T, the adatom diffusion rate ν_{d}, the adatom evaporation rate ν_{e}, and the step growth speed V_{s}, can be defined as following:

$$\nu_{\mathrm{d}} = \nu_0 \exp\left(-E_{\mathrm{diff}} / k_{\mathrm{B}} T\right)$$
$$\nu_{\mathrm{e}} = e_0 \exp\left(-E_{\mathrm{evp}} / k_{\mathrm{B}} T\right)$$
$$V_{\mathrm{s}} = F w$$

, in which ν_0 and e_0 are the diffusion and evaporation prefactors, E_{diff} and E_{evp} are the energy barriers for adatom diffusion and evaporation, and w is the width of the terrace. As the unit of the deposition flux is [ML s^{-1}] (or [atom per site per second]) and w is the width of the terrace in [site number] (see supplementary figure 2), the unit of V_{s} is [atoms per second]. The prefactors ν_0 and e_0 have the dimension of frequency and their exact value depends on the vibration entropies of the ground and the transition states of adatoms during the hopping or desorption process [3]. It can be shown for copper at room temperature that the prefactor ν_0 almost equals the vibrational frequency of the adatoms within their potential well. The latter is also known as the "attempt frequency". However, it should be noticed that this equality is not valid in general [2,3].

High mobility materials have large diffusion terms and, therefore, the step speed term becomes insignificant, unless one assumes unrealistically large deposition rates or terrace widths. Similarly, materials with low vapor pressure do have a negligible adatom evaporation term. Hence, for the typical film deposition conditions of Cu, Ag, or Au at room temperature, the step speed as well as evaporation term can be safely omitted in Eq. 9 [2, 4], leading to:

$$\frac{\partial \theta_n}{\partial t} = \nu_d \frac{\partial^2 \theta_n}{\partial n^2} + F \qquad (10)$$

For the steady state situation with constant deposition flux, i.e. $\partial \theta_n / \partial t = 0$, the second order linear differential equation, Eq.10, has the solution of the type:

$$\theta_n = -\frac{Fn^2}{2\nu_d} + c_1 n + c_2 \qquad (11)$$

At the position of ascending and descending steps the adatom density would be:

$$\theta_0 = c_2$$
$$\theta_w = -\frac{Fw^2}{2\nu_\mathrm{d}} + c_1 w + c_2 \qquad (12)$$

To determine the constants c_1 and c_2 we make use the two following boundary conditions: mass conversation dictates that the sum of the attachment and detachment rates at the ascending as well as the descending step edges should be equal to the diffusion currents toward theses steps.

The combination of attachment and detachment rates at the ascending ($n = 0$) and descending ($n = w$) step edge are:

$$J_0^{\mathrm{att/det}} = \nu_0 \exp\left(-\frac{E_{\mathrm{diff}} + E_{\mathrm{form}} + \Delta E_{\mathrm{att}}}{k_\mathrm{B} T}\right) - \theta_0 \nu_0 \exp\left(-\frac{E_{\mathrm{diff}} + \Delta E_{\mathrm{att}}}{k_\mathrm{B} T}\right)$$
$$J_w^{\mathrm{att/det}} = \theta_w \nu_0 s_0 \exp\left(-\frac{E_{\mathrm{diff}} + \Delta E_{\mathrm{ES}}}{k_\mathrm{B} T}\right) - \nu_0 s_0 \exp\left(-\frac{E_{\mathrm{diff}} + E_{\mathrm{from}} + \Delta E_{\mathrm{ES}}}{k_\mathrm{B} T}\right) \qquad (13)$$

, where E_{form} is the formation energy of an adatom (conceptually this is the energy difference of a surface with a step and a kink in comparison with the same surface, in which one took one atom from a kink site and placed it on the terrace), ΔE_{att} is the attachment barrier for adatoms to the ascending step, ΔE_{ES} is the Ehrlich-Schwoebel barrier for adatoms to overcome a descending step edge, and s_0 is a dimensionless correction pre-factor associated with the hopping over the step. To be more precise, $s = s_0 \exp\left(-\Delta E_{\mathrm{ES}} / k_\mathrm{B} T\right)$ is the ratio of the hop frequencies over step and terrace [5]. By defining

$$a = \exp\left(-\frac{\Delta E_{\text{att}}}{k_{\text{B}}T}\right),\ \ s = s_0 \exp\left(-\frac{\Delta E_{\text{ES}}}{k_{\text{B}}T}\right),\ \ \nu_{\text{d}} = \nu_0 \exp\left(-\frac{E_{\text{diff}}}{k_{\text{B}}T}\right),\ \ \theta_{\text{eq}} = \exp\left(-\frac{E_{\text{form}}}{k_{\text{B}}T}\right) \quad (14)$$

Eqs. 13 can be simplified as:

$$\begin{aligned} J_0^{\text{att/det}} &= \nu_{\text{d}} \cdot a \cdot \theta_{\text{eq}} - \theta_0 \cdot \nu_{\text{d}} \cdot a \\ J_w^{\text{att/det}} &= \theta_w \cdot \nu_{\text{d}} \cdot s - \nu_{\text{d}} \cdot s \cdot \theta_{\text{eq}} \end{aligned} \quad (15)$$

The diffusion current at any point on the terrace can be calculated as:

$$J_n^{\text{diff}} = -\nu_{\text{d}} \frac{\partial \theta_n}{\partial n} = Fn - c_1 \nu_{\text{d}} \quad (16)$$

By combining Eqs. 15 and 16 the boundary conditions $J_0^{\text{att/det}} = J_0^{\text{diff}}$ and $J_w^{\text{att/det}} = J_w^{\text{diff}}$ can be written as:

$$\begin{aligned} \nu_{\text{d}} \cdot a \cdot \theta_{\text{eq}} - \theta_0 \cdot \nu_{\text{d}} \cdot a &= -c_1 \cdot \nu_{\text{d}} \\ \theta_w \cdot \nu_{\text{d}} \cdot s - \nu_{\text{d}} \cdot s \cdot \theta_{\text{eq}} &= Fw - c_1 \cdot \nu_{\text{d}} \end{aligned} \quad (17)$$

The combination of Eqs. 12 and 17 delivers the following relationship,

$$\begin{bmatrix} -\nu_{\text{d}} & 0 & \nu_{\text{d}} \cdot a & 0 \\ \nu_{\text{d}} & 0 & 0 & \nu_{\text{d}} \cdot s \\ 0 & 1 & -1 & 0 \\ w & 1 & 0 & -1 \end{bmatrix} \begin{bmatrix} c_1 \\ c_2 \\ \theta_0 \\ \theta_w \end{bmatrix} = \begin{bmatrix} \nu_d \cdot a \cdot \theta_{\text{eq}} \\ Fw + \nu_{\text{d}} \cdot s \cdot \theta_{\text{eq}} \\ 0 \\ Fw^2 / 2\nu_{\text{d}} \end{bmatrix},$$

, in which the four unknown parameters can be calculated as:

$$\begin{aligned} c_1 &= \frac{Fwa(sw+2)}{2\nu_{\text{d}}(asw+a+s)} \\ \theta_0 = c_2 &= \theta_{\text{eq}} + \frac{Fw(sw+2)}{2\nu_{\text{d}}(asw+a+s)} \\ \theta_N &= \theta_{\text{eq}} + \frac{Fw(aw+2)}{2\nu_{\text{d}}(asw+a+s)} \end{aligned} \quad (18)$$

By combining Eqs. 11 and 18, the adatom density at any point on the terrace can be deduced as:

$$\boxed{\theta_n = \theta_{\text{eq}} + \frac{Fw(an+1)(sw+2)}{2\nu_{\text{d}}(asw+a+s)} - \frac{Fn^2}{2\nu_{\text{d}}}} \quad (19)$$

Note that Eq. 19 produces the thermodynamically correct value for the adatom density on a terrace if the surface receives no deposition flux $F = 0$:

$$\theta = \theta_{eq} = \exp\left(\frac{-E_{\text{form}}}{k_{\text{B}}T}\right) \qquad (20)$$

To provide insight in the adatom density variation on terraces, we calculated the density profile on Cu(111) terraces for different widths (w) and the presence as well as absence of an Ehrlich-Schwoebel barrier (ΔE_{ES}), see supplementary figure 3. For this calculations we used the well-established, experimentally determined barriers for Cu(111): $\nu_0 = 10^{12}\ \text{Hz}$, $E_{\text{diff}} = 0.040\,\text{eV}$ [6], $s_0 = 15$ [2], $\Delta E_{\text{ES}} = 0.224\ \text{eV}$ [7], $\Delta E_{\text{att}} = 0\ \text{eV}$ ($\Delta E_{\text{att}} \approx 0\ \text{eV}$ for most metals at room temperature), and $E_{\text{form}} = 0.714\ \text{eV}$ [8]. Notice that two cases describe the situation of funneling at the GB's with effective terrace widths $w \le 20$ and $\Delta E_{\text{ES}} = 0.0\ \text{eV}$ ($s = 1$); see the main text for more explanation.

According to Gibbs-Thompson a macroscopic surface curvature change alters the attachment/detachment rates [2] and, hence, also the adatom densities θ_n on the terraces. For a polycrystalline gold film, which first has been brought to its equilibrium state at 750 K 9], it has been shown that upon starting the deposition at room temperature the surface roughness/curvature increase initially before it approaches a steady state value 10]. However, the time scale of the roughness development is significantly longer than that of the reversible stress jumps. Note that the surface curvature variations involve changes in the step and kink densities and their distributions, which is expected to happen much slower than the adatom density variations (refer to the main manuscript).

Moreover, although not mentioned in 10], after the deposition has been stopped, no significant decrease of the roughness towards the equilibrium state was observed, e.g. due to the decay of 2D islands on the mounds. This is due to the rather limited activation of the surface annealing processes at room temperature [11]. For our study, we, therefore, safely omit these additional correction terms associated with surface curvature variations.

Supplementary Note 3 - Chemical potential of a grain as a function of stress

Supplementary figure 5 shows a simplified model of a grain with thickness L_0 that contains already an internal pre-stress σ_g. For pedagogical reason we first consider σ_g as a hydrostatic stress, before we (below) address the more realistic case of a film under biaxial stress. The chemical potential of (and also within) this grain can be defined as the partial derivative of its free energy with respect to the amount of atoms, N, in the grain. Adding a (thin) layer to this grain with a certain thickness *t* that contains N_t atoms causes strain $\varepsilon = t / L_0$, which consequently rises the (potential) energy of the system by the amount of involved work W_t. Therefore, the chemical potential of a grain can be written as:

$$\mu_g = \left(\frac{\partial U}{\partial N} \right)_{N_t=0} = \lim_{t\to 0} \frac{\partial W_t}{\partial N_t} \qquad (21)$$

The applied work can be, in general, calculated as:

$$W_t = -\int_0^t \left(\sigma(\varepsilon) \cdot A \right) dL \qquad (22)$$

, in which σ is the stress in the grain that is a function of the strain ε, and L is the amount of displacement of the grain boundary. Please note that $\sigma(\varepsilon)$ is linear in the elastic regime of the material, but becomes highly non-linear once one passes this regime.

Let us start here with the simple case of the elastic regime, in which the stress is linearly related to the strain via the Young's modulus:

$$\sigma = E_0 \varepsilon \qquad (23)$$

In this case the work in Eq. 22 can be calculated as:

$$W_t = -\int_0^t \left(\sigma_g + E_0 \frac{L}{L_0} \right) A dL = -A \left[\sigma_g L + \frac{E_0}{L_0} \frac{L^2}{2} \right]_0^t = -A\sigma_g t - A \frac{E_0}{L_0} \frac{t^2}{2} \Rightarrow$$

$$W_t = -\sigma_g \Omega N_t - \frac{E_0}{AL_0} \frac{(\Omega N_t)^2}{2} \qquad (24)$$

, in which Ω describes the atomic volume of the material. As we assumed the film to be in the elastic regime, the pre-stress of the grain before the addition of the extra (thin) layer with thickness t is given by $\sigma_g = E_0 \varepsilon_{pre}$, if ε_{pre} describes the pre-deformation of the grain. By combining Eqs. 21 and 24:

$$\mu_{\mathrm{g}} = \lim_{t\to 0}\frac{\partial W_t}{\partial N_t} = \lim_{t\to 0}\left(-\sigma_{\mathrm{g}}\Omega - \frac{E_0}{AL_0}\Omega^2 N_t\right) = \lim_{t\to 0}\left(-E_0\varepsilon_{\mathrm{pre}}\Omega - \frac{E_0}{L_0}\Omega t\right) \Rightarrow$$

$$\boxed{\mu_{\mathrm{g}} = -E_0\varepsilon_{\mathrm{pre}}\Omega = -\sigma_{\mathrm{g}}\Omega} \qquad (25)$$

The generalization to the plastic regime (i.e. σ_{g} larger than the yield strength of the material) follows simply by the fact that the slope of the stress-strain curve, E, decreases to values less than the Young's modulus E_0 and, in addition, becomes strain dependent. Hence, for infinitesimal values of *t,* the applied work is again calculated as:

$$W_t = -\int_0^t \left(\sigma_{\mathrm{g}} + E\frac{L}{L_0}\right) A dL = -\sigma_{\mathrm{g}}\Omega N_t - \frac{E}{AL_0}\frac{(\Omega N_t)^2}{2} \qquad (26)$$

, and similarly the chemical potential is derived as:

$$\mu_{\mathrm{g}} = \lim_{t\to 0}\frac{\partial W_t}{\partial N_t} = \lim_{t\to 0}\left(-\sigma_{\mathrm{g}}\Omega - \frac{E}{L_0}\Omega t\right) \qquad (27)$$

Note that the $\partial W_t / \partial N_t$ term in the definition of chemical potential not only depends on the pre-stress of the grain, but also on the local elasticity at particular stress point of interest (local slope in the strain curve) as well as the t / L_0 ratio. Based on this insight, one can also derive the variation of the chemical potential that occurs on the basis of two different stress values:

$$\mu_2 - \mu_1 = \lim_{t\to 0}\left(-\sigma_2\Omega - \frac{E_2}{L_0}\Omega t\right) - \lim_{t\to 0}\left(-\sigma_1\Omega - \frac{E_1}{L_0}\Omega t\right) = \lim_{t\to 0}\left(-(\sigma_2 - \sigma_1)\Omega - \frac{(E_2 - E_1)}{L_0}\Omega t\right) \Rightarrow$$

$$\mu_2 - \mu_1 = -(\sigma_2 - \sigma_1)\Omega \quad \Rightarrow \quad \boxed{\Delta\mu_{\mathrm{g}} = -\Delta\sigma_{\mathrm{g}}\Omega} \qquad (28)$$

This leads to an important conclusion: the variation of the chemical potential of a grain is only a function of internal stress variation and, therefore, fully independent of the elasticity, the shape of the grain, and whether the material is in the elastic or plastic regime.

As mentioned above, Eq. 28 holds only for hydrostatic stress conditions. Let us now derive the biaxial stress case, as thin films are typically anchored to the surface in the in-plane directions while being unconfined in the out-of-plane direction. For the general case, the chemical potential is related to the stress and strain fields as [12]:

$$\frac{\partial \mu}{\partial \boldsymbol{\sigma}_{ij}} = -\boldsymbol{\Omega}_{ij} \qquad (29)$$

, in which $\boldsymbol{\sigma}_{ij}$ is the stress tensor and $\boldsymbol{\Omega}_{ij}$ is the average strain volume tensor caused by the insertion of, e.g., an atom or molecule in the film. Eq. 29 can be integrated as:

$$\Delta\mu = -\int_{\boldsymbol{\sigma}_{rs}^{0}}^{\boldsymbol{\sigma}_{rs}^{0}+\Delta\boldsymbol{\sigma}_{rs}^{\mathrm{rev}}} \boldsymbol{\Omega}_{ij} d\boldsymbol{\sigma}_{ij} \qquad (30)$$

, in which $\Delta\mu$ is the chemical potential variation corresponding to the reversible stress tensor $\Delta\boldsymbol{\sigma}_{rs}^{\mathrm{rev}}$ (r and s =1, 2, 3), $\boldsymbol{\sigma}_{rs}^{0}$ (r and s =1, 2, 3) represents the initial stress tensor in the film, and in which we are using Einstein's summation notation. For a general anisotropic reversible stress as well as strain volume tensor, the stress tensor can be diagonalized, and one can write:

$$\Delta\mu = -\Omega_{\mathrm{x}}\Delta\sigma_{\mathrm{x}}^{\mathrm{rev}} - \Omega_{\mathrm{y}}\Delta\sigma_{\mathrm{y}}^{\mathrm{rev}} - \Omega_{\mathrm{z}}\Delta\sigma_{\mathrm{z}}^{\mathrm{rev}} \qquad (31)$$

, in which Ω_{x}, Ω_{y}, and Ω_{z} are the Ω_{11}, Ω_{22}, and Ω_{33} components of the strain volume tensor. Note that here we have approximated the $\boldsymbol{\Omega}_{ij}$ to be independent of the stress in the film.

For an in-plane isotropic film under in-plane isotropic reversible biaxial stress, we have $\Delta\sigma_{\mathrm{x}} = \Delta\sigma_{\mathrm{y}} = \Delta\sigma_{\mathrm{g}}$ and $\Delta\sigma_{\mathrm{z}} = 0$, i.e.:

$$\Delta\boldsymbol{\sigma}_{rs}^{\mathrm{rev}} = \begin{bmatrix} \Delta\sigma_{\mathrm{g}} & 0 & 0 \\ 0 & \Delta\sigma_{\mathrm{g}} & 0 \\ 0 & 0 & 0 \end{bmatrix} \qquad (32)$$

Due to the in-plane isotropy in combination with the spherically symmetric volume of additional inserted atoms, also the strain volume becomes assumed isotropic, namely:

$$\boldsymbol{\Omega}_{ij} = \frac{1}{3}\Omega\boldsymbol{\delta}_{ij} = \begin{bmatrix} \Omega/3 & 0 & 0 \\ 0 & \Omega/3 & 0 \\ 0 & 0 & \Omega/3 \end{bmatrix} \qquad (33)$$

, in which $\boldsymbol{\delta}_{ij}$ is the Kronecker delta and the scalar Ω the atomic volume of the inserted atom [12]. By combining Eqs. 30, 31, and 32, the variation of the chemical potential of the film interior becomes:

$$\Delta\mu_{\mathrm{biaxial}} = -\frac{2}{3}\Omega\Delta\sigma_{\mathrm{g}} \qquad (34)$$

Supplementary References

1. Sandström R. & Hallgren, J. The role of creep in stress strain curves for copper. *J. Nuc. Mater.* **422,** 51-57 (2012).
2. Ibach, H. *Physics of Surfaces and Interfaces.* Chs. 4.3, 5.4, 10.1, 10.4, and 11.4 (Springer 2006)
3. Kürpick, U. Self-diffusion on (100), (110), and (111) surfaces of Ni and Cu: A detailed study of prefactors and activation energies. *Phys. Rev. B* **64,** 075418 (2001).
4. Michely, T. & Krug, J. *Islands, Mounds and Atoms.* Ch. 4.3 (Springer, 2004).
5. Giesen, M. & Ibach, H. Step edge barrier controlled decay of multilayer islands on Cu(111). *Surf. Sci.* **431,** 109-115 (1999).
6. Knorr, N. et al. Long-range adsorbate interactions mediated by a two-dimensional electron gas *K. Phys. Rev. B* **65,** 115420 (2002).
7. Giesen, M., Schulze Icking-Konert, G. & Ibach, H. Fast decay of adatom islands and mounds on Cu(111): a new effective channel for interlayer mass transport. *Phys. Rev. Lett.* **80,** 552-555 (1998).
8. Stoltze, P. Simulation of surface defects. *J. Phys.: Condens. Matter* **6,** 9495-9517 (1994).
9. Rost, M. J., Quist, D. & Frenken, J. W. M. Grains, growth, and grooving. *Phys. Rev. Lett.* **91,** 026101 (2003).
10. Rost, M. J. In situ real-time observation of thin film deposition: roughening, Zeno effect, grain boundary crossing barrier, and steering. *Phys. Rev. Lett.* **99,** 266101 (2007).
11. Giesen, M. & Ibach, H. Step edge barrier controlled decay of multilayer islands on Cu(111). *Surf. Sci.* **431,** 109-115 (1999).
12. Eliaz, N. & Banks-Sills, L. Chemical Potential, Diffusion and Stress - Common Confusions in Nomenclature and Units. *Corros. Rev.* **26,** 87-103 (2008).
